# Case Report: Robotic-assisted laparoscopic primary repair for pancreaticoduodenal grade V injury in a pediatric patient

**DOI:** 10.3389/fped.2026.1693462

**Published:** 2026-02-24

**Authors:** Zijian Liang, Xinxing Wang, Menglong Lan, Xiaogang Xu, Jixiao Zeng

**Affiliations:** 1 Department of Pediatric Surgery, Guangzhou Women and Children’s Medical Center, Guangzhou Medical University, Guangzhou, Guangdong, China; 2 Department of Pediatric Surgery, Zhujiang Hospital, Southern Medical University, Guangzhou, Guangdong, China

**Keywords:** duodenal injury, minimally invasive surgery, pancreatic injury, pediatrics, robotic surgery

## Abstract

**Background:**

High-grade pancreatic injury is rare but associated with significant mortality and morbidity. There is no consensus on whether nonoperative or operative treatment could result in good clinical outcomes. Minimally invasive surgery has been introduced to manage cases of abdominal trauma, but no application in high-grade pancreaticoduodenal injury has been reported.

**Case presentation:**

An 8-year-old boy complained of severe abdominal pain after a bicycle injury. On admission, the patient was hemodynamically stable with elevated serum amylase and lipase levels. Thoracoabdominal computed tomography revealed massive disruption of the pancreatic head with pancreatic duct disruption and gas accumulation in the posterior part of the pancreas indicating localized duodenal perforation. Surgery was indicated and our surgical team chose to perform in a minimally invasive way with robotic assisted laparoscopic system based on the previous successful experience. A massive disruption of the pancreatic head (AAST-OIS grade V in pancreatic injury) and a laceration involving 30% of the circumference of the first part of the duodenum (AAST-OIS grade II in duodenal injury) without biliary system involvement were confirmed during surgery. A partial resection of the devitalized pancreatic head, primary suture of the duodenal laceration, and Roux-en-Y pancreaticojejunostomy with preservation of the pancreatic tail was performed in robotic-assisted laparoscopy. The patient resumed oral feeding on postoperative day 10 and was discharged 14 days postoperatively. At the 1-year follow-up, the patient demonstrated satisfactory recovery without any significant complications.

**Conclusion:**

For hemodynamically stable patients with high-grade pancreaticoduodenal injury, operative management is an appropriate therapeutic strategy. Application of robotic surgery may be a potentially optimal choice for primary repair in pediatric severe pancreaticoduodenal injury.

## Introduction

Traumatic blunt pancreatic and duodenal injuries (PI and DI) are infrequent with the estimated incidence ranging from 0.19% to 0.7% of all trauma admissions (1–3). With motor vehicle and bicycle accidents being the most frequent causes, blunt trauma is the most common cause among both adult and pediatric patients (4). Complications such as acute hemorrhage, pancreatic leaks, abscesses, fistulae, and pancreatitis have been reported in pancreaticoduodenal injuries (PDI) and are associated with high mortality in severe cases (5, 6), with the reported overall mortality rate between 5% and 14% (2, 5, 7). Accurate diagnoses of these injuries are difficult due to the deep anatomical position and the fact that pancreatic damage usually presents with a delay (8). Therefore, imaging examinations, mainly computed tomography (CT) and magnetic resonance cholangiopancreatography (MRCP), are routinely used for diagnosing PDI and further evaluation to determine duct integrity and severity of injury (4, 9).

A management algorithm of patients with PDI based on the American Association for the Surgery of Trauma (AAST) Organ Injury Scale (OIS) (4) is widely accepted by most surgeons. However, management of high-grade PDI (AAST-OIS grade III–V) is still controversial in terms of both surgical indications and options, especially in pediatric patients. In the current study, we present a pediatric case with Grade V PDI treated with robotic-assisted laparoscopic primary duodenal repair, partial pancreatectomy for removal of the damaged pancreatic head, and reconstruction with Roux-en-Y pancreatojejunostomy, and share our experience in managing this rare kind of PDI.

## Case presentation

**Figure 1:**
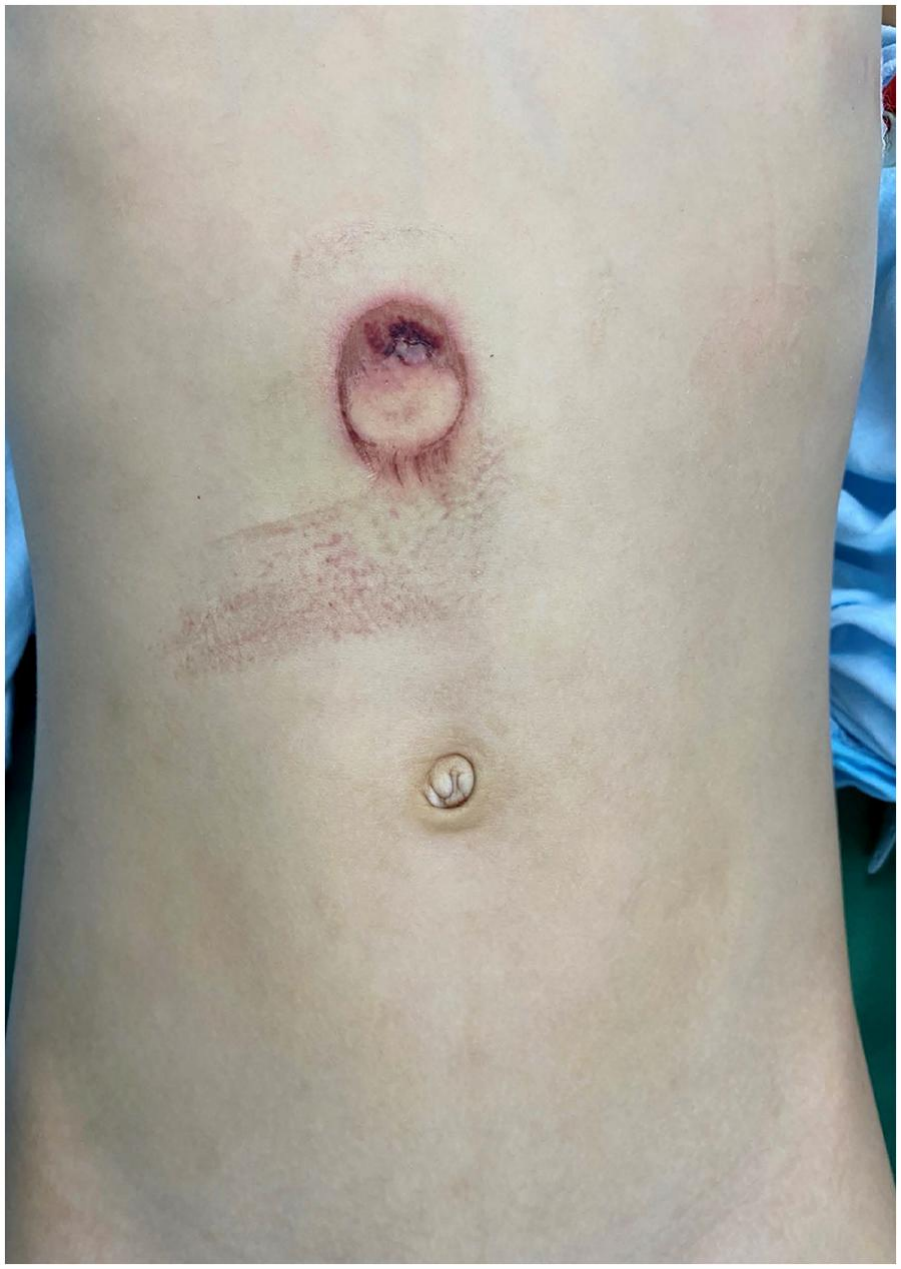
A circular bruise on the upper mid-abdomen, approximately 3 cm in diameter, caused by hitting the bicycle handlebar.

**Figure 2:**
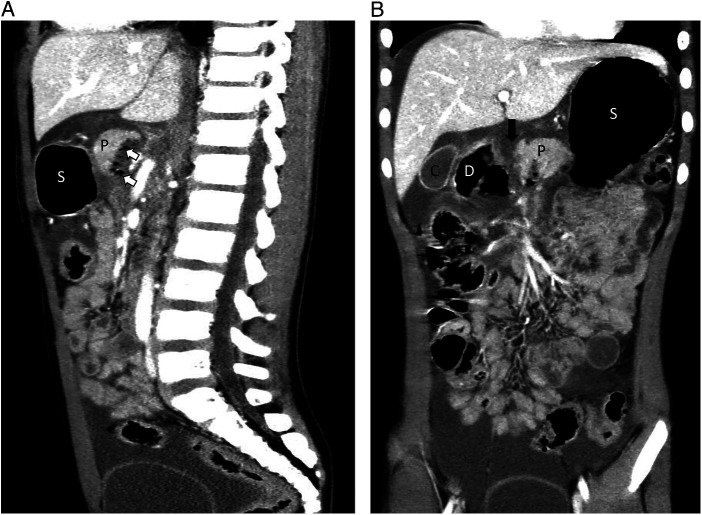
Imaging examination identified pancreatic injury with disruption of the pancreatic main duct and duodenal perforation (part I). **(A,B)** A transection (black arrow) at the junction between the head and the body of the pancreas, right to the superior mesenteric vein, with a small amount of gas accumulation in the posterior part of the pancreas (white arrows). D, duodenum; P, pancreas; C, cholecyst; S, stomach.

**Figure 3:**
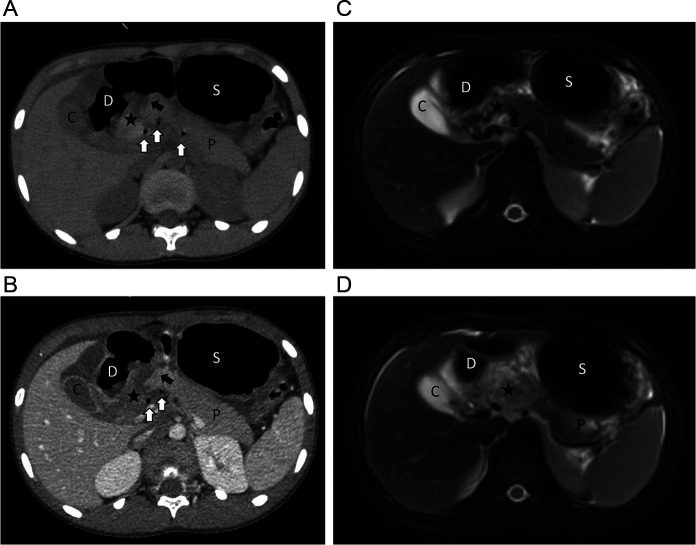
Imaging examination identified pancreatic injury with disruption of the pancreatic main duct and duodenal perforation (part II). **(A–D)** Pancreatic contusion (star mark) and a transection (black arrow) at the junction between the head and the body of the pancreas, right to the superior mesentery vein, with pancreatic head hematoma and a small amount of gas accumulation in the posterior part of the pancreas (white arrows). Thickening and swelling of the duodenal wall indicated duodenal contusion and localized perforation. D, duodenum; P, pancreas; C, cholecyst; S, stomach.

An 8-year-old boy was admitted to a nearby hospital because of severe abdominal pain experienced after a bicycle handlebar hitting on the upper abdomen during a crash. Twenty-nine hours after the initial injury, the boy was transferred to our medical center, a tertiary pediatric emergence center. On arrival, the patient was conscious, but complained of constant abdominal pain with hematemesis. Primary physical examination revealed tenderness with a circular bruise on the upper mid-abdomen, approximately 3 cm in diameter (Figure 1). The patient was hemodynamically stable (pulse rate 130 min, blood pressure 119/79 mm Hg). Laboratory tests revealed elevated serum levels of amylase and lipase (serum amylase: 320 U/L, lipase: 543 U/L). Ultrasound examination showed bruising of the head of the pancreas and turbid pelvic fluid. Radiography showed some localized free gas accumulation in the right upper abdomen. Thoracoabdominal CT scan further identified pancreatic contusion and a transection at the junction between the head and the body of the pancreas, right to the superior mesentery vein, with pancreatic head hematoma and a small amount of gas accumulation in the posterior part of the pancreas (Figures 2A,B, 3A,B). Additionally, thickening and swelling of the duodenal wall were observed, with peripheral gas accumulation indicative of duodenal contusion and localized perforation. The CT scan also revealed abdominopelvic and gallbladder fossa effusions. To further assess the integrity of the main pancreatic duct, emergency MRCP was performed. The MRCP findings were consistent with the CT results and confirmed a rupture of the main pancreatic duct (Figures 3C,D). Based on the clinical manifestations and ancillary tests, a diagnosis of rupture of the pancreatic duct in the pancreatic head and a duodenal perforation was provisionally established and operative treatment was indicated. Robotic-assisted laparoscopy for minimally invasive procedure was a priority in our surgical team based on the previous experiences in dealing with complex pancreas diseases, such as pancreatic head tumor.

**Figure 4:**
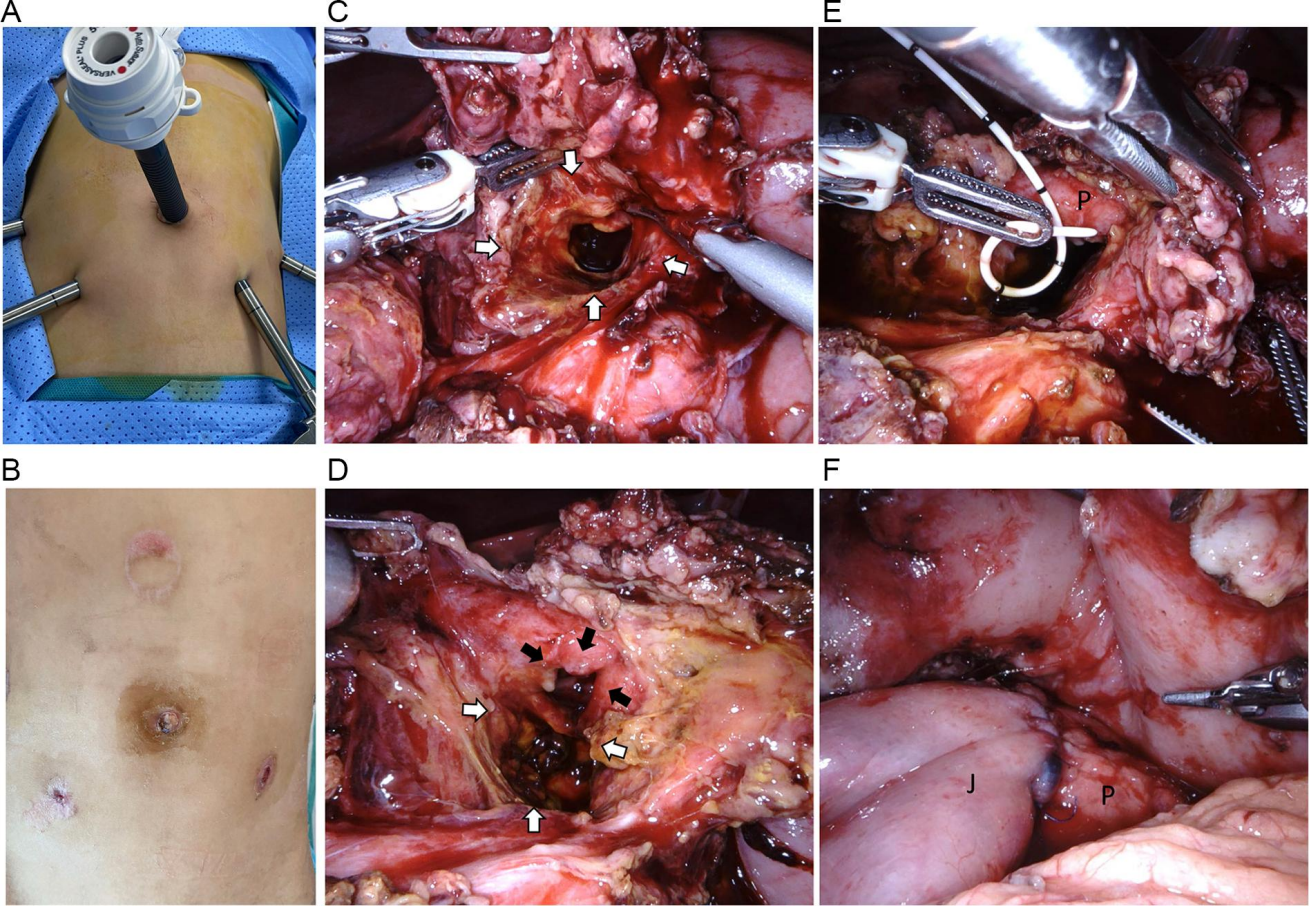
Findings during the surgery and the important steps in surgical procedure. **(A)** Docking of robotic-assisted surgery. **(B)** Postoperative abdominal appearance. **(C)** The crash tunnel through the abdominal wall deep into the duodenum (white arrows). **(D)** D1 laceration (3 cm in length and involving 30% of the circumference), identifying AAST-OIS grade II duodenal injury (black arrows). **(E)** Stent in the main pancreatic duct after the debridement of the damaged pancreatic head. **(F)** The jejunopancreatic anastomosis. J, jejunum; P, pancreas.

A five-port approach was performed with minimally invasive wounds sized in 5–12 mm (Figures 4A,B). Intraoperative findings confirmed a massive disruption of the pancreatic head (AAST-OIS grade V in PI) and a laceration 30% of the circumference of the first portion of the duodenum (duodenal bulb, D1) (AAST-OIS grade II in DI) without biliary system involvement (Figures 4C,D). A partial resection of the devitalized pancreatic head, primary suture of the duodenal laceration, and Roux-en-Y pancreaticojejunostomy with preservation of the pancreatic tail was performed using the laparoscopic approach with the da Vinci Xi Surgical System (Figures 4E,F). Intraoperative indocyanine green fluorescence imaging facilitated recognizing the devitalized tissue and ensuring a complete debridement. The operation lasted 11 h, with an estimated blood loss of 50 mL. Intraoperatively, the patient received a transfusion of 1 unit of packed red blood cells. Postoperatively, the patient was admitted to the intensive care unit for 3 days. The level of amylase and the amylase-to-serum amylase ratio gradually decreased to the normal range one week after the surgery. Four external drainages were placed, namely at the site of duodenal repair, pancreaticojejunostomy, transection of the pancreatic head, and the pelvic cavity. They were removed 10–14 days postoperatively when the amount of drained fluid per drainage remained less than 20 mL for 3 days. At the same time, the volume of gastric fluid diminished and the nasogastric tube was removed 10 days postoperatively. The patient resumed oral feeding on postoperative day 10 and was discharged 14 days postoperatively.

At the 1-year follow-up, the patient demonstrated satisfactory recovery without significant complications. Repeated ancillary tests were performed during every routine outpatient visit postoperatively. Serum levels of amylase and lipase remained within normal range, and ultrasound scan of the upper abdominal cavity showed no pancreatic fistula, cyst, or atrophy during the most recent follow-up. In addition, endocrine function of the pancreas was maintained, considering that normal levels of glucagon and insulin were detected in the serum. We ordered no further radiological examination such as CT or MRCP as no abnormal results were found during the follow-up.

## Discussion

In this report, we presented the application of robotic-assisted laparoscopic partial resection of the massively disrupted pancreatic head, pancreatojejunostomy, and duodenal repair for grade V PDI. This is the first documented application of this approach for managing grade V PDI, both in pediatric and adult patients. In addition, we shared our experience and discussed the detailed surgical technique in dealing with complex pancreatoduodenal trauma.

In this case, a massive disruption of the pancreatic head (AAST-OIS grade V in PI) and a laceration involving 30% of the circumference of D1 (AAST-OIS grade II in DI) without biliary system involvement were confirmed during surgery. Our surgical team took three steps in deciding about the final surgical procedure, as detailed below. First, as the patient was hemodynamically stable, damage control procedure was not the optimal choice, given that the principles in management of high-grade PDI are to curtail bleeding, ensure removal of entire devitalized tissue and reconstruct the function of the pancreas, biliary system, and gastrointestinal tract (10). Based on these surgical principles, our surgical team considered the primary repair as the optimal choice. Second, we found errhysis in the massively disrupted pancreatic head forming a large retroperitoneal hematoma, and there was no biliary system involvement as no bile-like exudation was found. Therefore, we undertook a partial resection of the pancreatic head and duodenal repair instead of pancreatojejunectomy to stop errhysis and remove the devitalized pancreas in a less invasive way. Viable uninjured pancreatic tissue should be left alone whenever possible in all types of pancreatic injuries to preserve primary organ function and decrease short- and long-term morbidity (11). Third, an end-to-end invagination pancreaticojejunostomy with Roux-en-Y reconstruction (12–14) was performed. Compared with duct-to-mucosa anastomosis (14), which achieves excellent results in adults (15), this technique can avoid difficulty in anastomosis with the main pancreatic duct, which is small and fragile in pediatric patients and easy to be torn during the suture (15, 16). Both our previous (12) and current study demonstrate that the end-to-end invagination technique was safe and no severe complication was found using this technique in children.

Before a proper approach to a PDI is decided, AAST-OIS grade of the injury, patient stability, and the severity of concomitant life-threatening injuries should all be taken into consideration (6). Damage control is applied for patients with severe injury in neighboring organs or unstable vital signs despite proper fluid management (17). Definitive management should be undertaken when physiological stabilization has been achieved.

The management algorithm of adult patients with PDI is mostly based on AAST-OIS (4). Nonoperative management (NOM) should be considered for AAST grade I or II with hemodynamic stability and no associated injury (4, 6). Distal pancreatectomy is recommended for AAST grade III injury of the distal half of the pancreas, and formal pancreaticoduodenectomy is advised in cases with AAST grade IV or V injuries (4, 6). However, these recommendations are based on trauma in adult patients, and there are no specific guidelines for pancreatic trauma in pediatric patients (18). As a common view in pediatric surgery, conservative management of solid-organ trauma was pioneered in pediatric trauma (18). The reasons are as follows: (1) to avoid future organ insufficiency from partial or complete organ resection; (2) to reserve a natural anatomy as reconstruction surgery is technically difficult in children because of their small size of anatomy elements, which may potentially result in postoperative complications, including leaks and fistula (8, 19, 20). Therefore, the current surgical trend has been moving towards conservative treatment for both low- and high-grade PDI (8, 19, 20). Only children with multivisceral injuries or continued hemorrhagic shock after initial blood transfusion are being considered for laparotomy in these years (21).

Based on the above principle for NOM, studies of NOM in pediatric PDI have recently been published. Although the overall success rate of initial NOM is high, ranging from 87% to 100% in pediatric PDI (21–24), the heterogeneous population with different AAST grades may have led to potential bias in these studies. For example, in a retrospective study involved 51 pediatric patients, there were only nine patients in the high-grade group (23). With a large heterogeneous population in such situation, a subgroup analysis of the success rate could be more precise for supporting the NOM treatment in high-grade PDI.

In addition, NOM is believed to lead to late pancreatic complications (18), resulting in more repeat interventions and longer time to complete resolution of high-grade pancreatic injury in the NOM group compared with the operation management (OM) group in children (24, 25). For example, pseudocysts of the pancreas, which are one of the most common complications in PDI, happen in 18%–47% in NOM patients (1, 20, 22–25) and sometimes require late surgical interventions such as open drainage, Roux-en-Y cystojejunostomy, or cystogastrostomy (1, 21, 26). Among the 24 patients with pancreatic pseudocysts after PDI, Zhang et. reported that half required external drainage (23). Another complication associated with NOM for high-grade PDI is pancreatic atrophy distal to the transection. A study on long-term outcomes over 10-year follow-up after NOM for high-grade PDI (AAST-OIS grade III–IV) showed a frequent occurrence of pancreas body/tail atrophy on follow-up CT scans (21, 24). The atrophy of the pancreas could be related to the disruption of the pancreatic duct (27), but it was not associated with the damage to the pancreatic function, given that no pancreatic insufficiency was found in any of the cases that showed gland atrophy (21, 24). However, the cause and effect of the gland atrophy after pancreatic injury are still unknown, and long-term follow-ups for these patients into adulthood are needed (21, 24). In conclusion, it would be still too early to draw any conclusion as to whether NOM or OM is optimal for managing high-grade PDI in children.

Recent literature has highlighted the usefulness and safety of minimally invasive technique in the cases with abdominal trauma (28–30). Compared with laparotomy, laparoscopic surgery is associated with quicker recovery, less pain, shorter hospital stay, and lower morbidity and mortality, with favorable wound appearance and lower rate of wound complications (29, 30). Experts also advocate procedures with robotics for emergency general surgery for hemodynamically stable patients (28). Although inconsistent results have been found in recent comparative studies between robotic and laparoscopic surgery in abdominal trauma (31–33), the robotic system provides a wide three-dimensional field of vision, flexible tools, and the elimination of physiological tremors. Thus, it allows better control of hemostasis and precise dissection of tissues and hard sutures, facilitates the performance of some difficult procedural steps, and reduces the risk of conversion and complications (28, 34).

To date, there have been limited documented cases of robotic-assisted emergency surgery in both adults and children, mainly addressing cholecystectomy, appendectomy, bowel resection, lysis of adhesion, hiatal hernia, and repair of perforated gastrojejunal ulcers (31–33, 35). Our study is the first documented report representing the application of robotic-assisted laparoscopic partial resection of the massively disrupted pancreatic head, pancreatojejunostomy, and duodenal repair for grade V PDI. This is a landmark for the robotic laparoscopy application in pediatric PDI, which represents one of the most complex abdominal traumas. The good recovery of our patient and the absence of severe complications demonstrate robotic-assisted laparoscopic surgery to be safe and efficacious even in children with severe abdominal trauma. Additionally, our successful attempt could broaden the spectrum of emergency surgery in a robotic laparoscopic approach, which could encourage more attempts in selected similar cases.

Our study has certain limitations. The clinical result in our reported case may be limited to robotic laparoscopy application in pediatric PDI. More high-quality comparative studies are needed to better evaluate the safety and efficacity. Moreover, the follow up was relatively short, and a prolonged observation is required to confirm the results of the present study.

## Conclusion

For hemodynamically stable patients with high-grade PDI, OM may be considered an appropriate therapeutic strategy. Application of robotic surgery may be a potentially optimal choice for primary repair in pediatric severe PDI.

## Data Availability

The raw data supporting the conclusions of this article will be made available by the authors, without undue reservation.
